# GP and nurses' perceptions of how after hours care for people receiving palliative care at home could be improved: a mixed methods study

**DOI:** 10.1186/1472-684X-8-13

**Published:** 2009-09-14

**Authors:** Heather M Tan, Margaret M O'Connor, Gail Miles, Britt Klein, Peter Schattner

**Affiliations:** 1 Palliative Care Research Team, School of Nursing and Midwifery, Monash University, Frankston, Victoria 3199, Australia; 2 Vivian Bullwinkel Chair of Palliative Care Nursing, Monash University, Victoria, Australia; 3 Royal District Nursing Service, Melbourne, Victoria, Australia; 4 National eTherapy Centre, Faculty of Life and Social Sciences, Swinburne University of Technology, Hawthorn, Victoria, Australia; 5 Department of General Practice, Monash University, Notting Hill, Victoria, Australia

## Abstract

**Background:**

Primary health care providers play a dominant role in the provision of palliative care (PC) in Australia but many gaps in after hours service remain. In some rural areas only 19% of people receiving palliative care achieve their goal of dying at home. This study, which builds on an earlier qualitative phase of the project, investigates the gaps in care from the perspective of general practitioners (GPs) and PC nurses.

**Methods:**

Questionnaires, developed from the outcomes of the earlier phase, and containing both structured and open ended questions, were distributed through Divisions of General Practice (1 urban, 1 rural, 1 mixed) to GPs (n = 524) and through a special interest group to palliative care nurses (n = 122) in both rural and urban areas.

**Results:**

Questionnaires were returned by 114 GPs (22%) and 52 nurses (43%). The majority of GPs were associated with a practice which provided some after hours services but PC was not a strong focus for most. This was reflected in low levels of PC training, limited awareness of the existence of after hours triage services in their area, and of the availability of Enhanced Primary Care (EPC) Medicare items for care planning for palliative patients. However, more than half of both nurses and GPs were aware of accessible PC resources.

Factors such as poor communication and limited availability of after hours services were identified the as most likely to impact negatively on service provision. Strategies considered most likely to improve after hours services were individual patient protocols, palliative care trained respite carers and regular multidisciplinary meetings that included the GP.

**Conclusion:**

While some of the identified gaps can only be met by long term funding and policy change, educational tools for use in training programs in PC for health professionals, which focus on the utilisation of EPC Medicare items in palliative care planning, the development of advance care plans and good communication between members of multidisciplinary teams, which include the GP, may enhance after hours service provision for patients receiving palliative care at home. The role of locums in after PC is an area for further research

## Background

Palliative care (PC) emerged as a discipline during the twentieth century [1] with notable advances made in Australia, beginning in the 1980s [2]. As health services provided for those 'whose disease is not responsive to curative treatment', the PC movement reached acute hospitals, the home and sub-acute settings such as aged care [3,4].

Another development has been the acceptance of the benefits of PC being delivered beyond those with a diagnosis of cancer to those people suffering from a chronic illness [3]. The Australian government has remained supportive of maintaining a person's optimal health status whilst living in the community [5].

The literature has established that PC service delivery has changed from a focus on institutional settings to home service style delivery [6,7] and from a single discipline service to a multidisciplinary, supportive service within all settings [8]. The necessity for GPs and primary care providers, such as nurses, to be educated and skilled in PC is also the focus of a number of articles [9,10]. There is, however, limited evidence of how PC after hours services are accessed by clients and carers when it is not part of a particular PC service. Support, anxiety and sleep problems have been reported as concerns in after hours telephone enquiries received by GPs and hospitals [11].

Rurality features significantly in the Australian literature and the significance of this setting for PC provision has been reported [12] with PC support services provided by differing models of care. Enhanced Primary Care, under Medicare (Australia's system of universal access to health care) is one of a number of initiatives for chronic disease management through a multidisciplinary team approach, which when extrapolated equates to the delivery of a palliative approach in community settings [13]. The recognition of the significant carer contribution required to maintain a dying person at home was at the centre of an Australian study of carer's physical and mental wellbeing [14].

The provision of 24 hours a day, seven days per week PC services has been advocated, [15] though the question arises as to how these PC services operate in rural and urban communities where limited availability has been reported. There is an expectation for all community services offering a palliative approach to be equitable in rural and urban communities. Twenty four hour access is described as the 'gold standard' for PC service delivery, yet little data is available to justify the cost of this initiative to health care planners [15]. The means of distribution of concise, relevant and timely information about unstable patients to nurses and GPs involved in after hours care is challenging [16]. One rural region of Northern Victoria (Australia) reported that due to the shortage of GPs, an after-hours PC rural telephone triage system added an extra burden to the nursing staff addressing PC enquiries [17]. This is the first published report of the extra burden on nursing staff for PC after hours services.

Access to after hours PC community services by people receiving PC and their carers, in both rural and urban environments of Victoria is the central focus of this Australian study conducted in three phases. Participants were drawn from three Divisions of General Practice, one urban, one urban and semi-rural and the other rural and remote. The findings of phase 1, a qualitative study of stakeholders' experience of after hours palliative care service provision [18], also support many of the findings described above and formed the basis for this mixed methods phase 2 of the project reported in this paper.

## Methods

### Participants

Only GPs (*n *= 524) who were affiliated with the participating Divisions of General Practice in Victoria and nurses (*n *= 112) who were members of the Palliative Care Special Interest Group of the Australian Nursing Federation (Victorian Branch) were invited to participate in this phase of the study.

### Study Instruments

Key findings of the qualitative phase 1 [18] of the project were utilised in the design of questionnaires, for GPs (see Additional file 1) and nurses (see Additional file 2), to facilitate further exploration of the challenges of providing after hours care for people receiving palliative care at home. These questionnaires consisted of six main sections which collected information about demographic details, service for which the participant worked, after hours services in their area, patient management, barriers to effective after hours care (9 items) and strategies to improve this care (13 items). The latter two sections included a five point Likert Scale (1-5) allocated as follows: "strongly agree" = 1, "agree" = 2, "disagree" = 3, "strongly disagree" = 4 and "don't know" = 5. Questionnaires were distributed to both GPs in the participating regions and nurses involved in palliative care provision.

All GPs registered with the participating Divisions of General Practice were emailed, by their Division secretary, alerting them to the study. Explanatory sheets and the questionnaires were then mailed, by the Divisions, to each GP along with a reply paid envelope. They registered their interest by completing and returning the questionnaire in the reply paid envelop provided. In the case of nurses, all members of the special interest group were emailed by the group leader, alerting them to the study and asking them to contact the project officer for the study to obtain more information and the questionnaire. These were mailed, along with a reply paid envelope to those who requested it. A reminder notice was sent via email to all the GPs and nurses one month after initial contact had been made by the relevant Divisions of General Practice and the PC Nurses Special Interest Group.

### Data Analysis

Data were coded, entered and analysed with the assistance of SPSS data analysis software version 16.0. Descriptive analysis using frequency distribution and mean scores was carried out. To compare GP's and nurse's responses to questions relating to 'factors which would impact on after hours PC service' and 'strategies which may improve after hours PC service', a comparative analysis was carried out.

### Ethics Approval

Ethics approval was granted by the Monash University Standing Committee on Ethics in Research Involving Humans.

## Results

### Response rates

Five hundred and twenty four GPs and 122 nurses were informed about the study. Of the above, 114 GPs (22% response rate) and 52 nurses (43% response rate) returned completed questionnaires. Participant demographic data including gender, years of experience, specialist PC training and reasons for not having any are outlined in Table 1. It was found that only 15% of participating GPs had undertaken post graduate PC training, 70% of those who had not citing lack of time as the reason for not doing so. 69% of participating nurses had undertaken postgraduate PC training, lack of time again being the dominant reason for not doing so.

**Table 1:** Sample characteristics of the participants (GPs and Nurses)

	**Total (n = 166)**		**GPs (n = 114)**		**Nurses (n = 52)**	
	**N**	**%**	**N**	**%**	**N**	**%**
**Gender**						
Male	80	48.2	76	66.7	4	7.7
Female	86	51.8	38	33.3	48	92.3

**Years Experience**						
0-5	7	4.2	7	6.1	0	0
6-10	6	3.6	5	4.4	1	1.9
11-20	55	33.1	38	33.3	17	32.7
20+	98	59.1	64	56.1	34	65.4

**PC Training**						
Yes	53	31.9	17	14.9	36	69.2
No	113	68.1	97	85.1	16	30.8

**Reason for no post grad. Training**	(n = 113)		(n = 97)		(n = 16)	
Lack of time	79	69.9	67	69.1	12	75.0
Lack of course	14	12.4	13	13.4	1	6.0
Lack of interest	N/A	-	12	12.4	N/A *	-
Other	8	7.1	5	5.1	3	19.0

*All nurse participants had declared their interested in palliative care by being member of this interest group

### Service provision

Data relating to the provision of after hours service, the use of Enhanced Primary Care Medicare items, the existence of a telephone triage service in their area, access to palliative care resources and level of satisfaction with service provision are shown in Table 2.

**Table 2:** Service provision in their service/area frequency (percentage) data

	**Total (n = 166)**		**GPs (n = 114)**		**Nurses (n = 52)**	
	**N**	**%**	**N**	**%**	**N**	**%**

**After Hours Service Provision by their Service**						
Calls	102	61.4	68	59.6	34	65.3*
Visits	122	73.5	80	70.2	42	80.7

**EPC Items**						
Use generally	N/A		84	73.7	N/A**	
Aware can use for PC planning	N/A		60	52.6	N/A**	

**Tel. Triage Protocol in Area**						
Yes	50	30.1	16	14.0	34	65.4
No	52	31.3	42	36.9	10	19.2
Don't Know	64	38.6	56	49.1	8	15.4

**Aware of PC Resources**						
Yes	102	61.5	67	58.8	35	67.3
No	64	38.5	47	41.2	17	32.7

**Current Services** **Satisfactory**						
(agree or strongly agree combined)						
Hospital discharge planning	107	64.5	79	69.3	28	53.8
Home care planning	141	84.9	100	87.7	41	78.8
After hours service provision	123	74.1%	88	77.2	35	67.3

Note *8 nurse participants did not currently work in PC service; * *Enhanced Primary Care Items (EPC) not available to nurses

Some GPs indicated that they did not use the EPC items because they do not like the paper work involved. Some also said that they would find it useful to have more information about what PC services are available in their area and what they can provide. Additional resources such as more access to specialist PC consultants and PC nurses, patient data after hours and to RDNS palliative care guidelines were suggested.

It should be noted that in most cases in urban and semi-rural areas GP after hours service was provided by locums. Of the 83 respondents in the urban and mixed urban/semi-rural divisions only 14 indicated that there was no locum service and all of these were located in the urban/semi-rural division. However, only 2 of the 29 GP participants in the rural/remote division had access to a locum service.

### Factors impacting on after hours PC service

Participating GPs and nurses were also asked about factors which they considered may impact on effective after hours service provision. Table 3 shows the percentage of participants who strongly agreed or agreed with the given statement. Poor communication between nurses and GPs was considered the most important factor while, of the possibilities considered, poor phone coverage was the least important.

**Table 3:** Factors which may impact on after hours PC services as endorsed by GPs and Nurses (Percentage frequencies)

**Factors which may impact on after hours PC services**	**GPs**	**Nurses**
Poor communication between GPs & nurses	82%	85%
High cost of locum services	54%	67%
Patients unwilling to call after hours services	59%	62%
Nurses unsafe at night	41%	63%
Limited mobile phone coverage	40%	44%
Limited access to emergency medication	69%	71%
Limited availability of GPs after hours	76%	90%
Limited availability of PC nurses after hours	79%	83%
No access to interpreters after hours	49%	54%

Further analysis including mean, standard deviation and t test were undertaken comparing the responses of GPs and nurses to the questions in this section of the questionnaire. The results of this analysis are shown in Table 4. Note that lower scores equate to higher rating. Nurses and GPs held similar views on six of the nine factors which potentially impact on after hours PC. There were however three factors on which they held significantly different views nurses consistently rating them as more likely to be barriers to care. These were: nurses feeling unsafe at night, limited access to emergency medication after hours and limited availability of GPs after hours.

**Table 4:** Means, Standard Deviations and scores on the factors which may impact on after hours PC services(Mean scores)

	**GPs**			**Nurses**			
**Question**	**N**	**M**	**SD**	**N**	**M**	**SD**	***t *score**

Poor communication between GPs & nurses	112	1.73	.92	52	1.71	.83	.14

High cost of locum services	104	2.66	1.36	49	2.41	1.44	1.06

Patients unwilling to call after hours services	112	2.53	1.07	51	2.43	1.10	.53

Nurses unsafe at night	111	2.81	1.09	52	2.33	1.00	2.70*

Limited mobile phone coverage	111	2.84	1.15	52	2.56	1.20	1.43

Limited access to emergency medication	112	2.24	.92	52	1.88	.88	2.34**

Limited availability of GPs after hours	112	2.09	1.13	51	1.61	.94	2.66*

Limited availability of PC nurses after hours	112	2.08	1.14	51	1.82	.93	1.41

No access to interpreters after hours	110	2.98	1.43	49	2.63	1.37	1.44

*p < 0.01; ** p < 0.05

### Strategies to Improve After Hours PC Services

Participants were invited to rate suggested strategies which may improve after hours services. Table 5 indicates the percentage of participants who either strongly agreed or agreed (categories combined) with the suggested strategy. Individual patient protocols, accessible to after hours staff and PC trained respite carers were the two strategies considered most likely to improve after hours service provision by both GPs and nurses. On the other hand the allocation of more specific PC beds in local hospitals and the referral of patients to PC services at time of diagnosis were considered by both GPs and nurses to be least likely to improve after hours PC services.

**Table 5:** GPs and Nurses views of strategies which may improve after hours PC service using percentage frequencies

**Strategies that may improve after hours PC**	**GPs**	**Nurses**
Standardised written protocol	86%	87%

Individual patient protocol	91%	88%

Formal protocol between PC service & indigenous Australians	63%	87%

Regular multidisciplinary meetings	70%	92%

Referral to PC service at time of diagnosis	69%	63%

More nurses for home visits	82%	81%

PC trained nurse on after hours tel. service	86%	92%

PC trained respite carers	90%	94%

More support and debriefing for GPs/nurses	76%	87%

More support for carers	58%	96%

More specific PC beds in local hospitals	54%	23%

Greater access to equipment for home care	83%	83%

Legislation to allow nurse evaluation of death	58%	87%

Further analysis to examine the differences between the two groups -including mean, standard deviation and *t *test - was undertaken. This revealed significant differences between the two groups on seven of the 13 suggested strategies for improvement of after hours PC. These seven strategies were: a formal protocol between PC service and indigenous Australians, regular multidisciplinary team meetings, more nurses for home visits, PC trained nurses on after hours telephone service, PC trained respite carers, more support and debriefing for GPs and nurses and legislation to allow nurse evaluation of death. The results of is analysis this shown in Table 6

**Table 6:** Mean, Standard Deviation and *t *scores on strategies which may improve after hours PC service between GPs and Nurses

	**GPs**			**Nurses**			
**Question**	**N**	**M**	**SD**	**N**	**M**	**SD**	**t**

Standardised written protocol	112	1.78	.91	51	1.53	.76	1.70

Individual patient protocol	112	1.75	.86	52	1.60	.82	1.09

Formal protocol between PC service & indigenous Australians	104	2.66	1.56	51	1.90	.99	3.19*

Regular multidisciplinary meetings	111	2.19	1.01	52	1.63	.74	3.53*

Referral to PC service at time of diagnosis	111	2.13	1.07	51	2.12	.95	.05

More nurses for home visits	110	2.09	1.18	52	1.67	.86	2.29**

PC trained nurse on after hours tel. service	111	1.80	.93	51	1.33	.55	3.33*

PC trained respite carers	110	1.79	.73	51	1.39	.57	3.44*

More support and debriefing for GPs/nurses	110	2.15	.96	52	1.73	.84	2.73*

More support for carers	111	1.89	.98	52	1.62	.57	1.90

More specific PC beds in local hospitals	77	2.01	1.24	16	1.44.	.51	1.82

Greater access to equipment for home care	107	1.91	.88	49	1.96	.96	-.34

Legislation to allow nurse evaluation of death	112	2.61	1.34	52	1.71	.83	4.40***

* p < 0.01; ** p < 0.05; ***p < 0.001

## Discussion

The findings of this phase of the project provided more detailed information about some of the main areas of concern regarding the provision of after hours palliative care that were identified in phase 1 of the project [18]. In particular these data present the views of two different groups of health professionals, GPs and palliative care nurses, regarding how after hours care of palliative patients in the community might be improved.

### Demographic Profile

The demographic details of the participants provided some points of interest. Almost all (GPs 89% and nurses 98%) who participated in the study had more than 11 years experience and in both cases the majority of those who responded had more than 20 years experience. If this is representative of the general GP and PC nurse work force, this has important implications for future palliative care services in the community as many of these people are approaching retirement age. In the case of GPs this is consistent with other findings [19], which identified that in a sample of GPs working in the southern and north western regions of Sydney, those not involved in providing PC were likely to be younger and have less GP experience than those who did provide this care. In our study the percentage of GPs who had undertaken postgraduate palliative care training (14%) was higher than that identified in the above Sydney study (9.1%) [19]. It should be noted that our finding of 69% of nurses having undertaken palliative care training is not representative of the general nursing population, as participants were all drawn from a PC nurses special interest group.

### Service provision

While many (more than 60%) of the participants were associated with practices or organisations which provided after hours PC services there was a notable lack of knowledge about service provision. For example, 47% of GPs were unaware that EPC Medicare items could be used for care planning for people receiving PC, both in the development of advance care plans and for team meetings with multidisciplinary PC teams, even though this information is provided on relevant Division of General Practice websites. It was also found that 49% of GPs did not know if after hours triaged telephone services were available in their area. One explanation for this may be that the majority of the participating GPs did not personally provide after hours services but relied on locum services, a notion supported by the data presented above in relation to the use of locum services. Clearly the availability of locum services is one of the inequitable situations in the provision of health care (and no doubt after hours palliative care) between urban and rural/remote areas. Phase 1 of this study also indicated that some of the problems experienced with after hours locum services included costs, lack of PC training among locum doctors, lack of knowledge of specific patient needs and very long delays in getting service [18]. Limited literature is available regarding provision of after hours PC services by locum GPs in Australia and so this offers opportunity for further investigation.

It was also observed that GPs (77%) considered that current after hours services were either very satisfactory or satisfactory (combined result) compared to only 67% of nurses who participated. Given that nurses participating in this study were recruited from a PC nurses' special interest group, this difference may be a reflection of greater nurse involvement in the provision of after hours PC services.

### Factors which may impact of after hours PC services

Both nurses and GPs were asked to indicate their agreement or otherwise with a number of statements about factors which may impact on after hours service provision. For six of the nine factors listed there was no significant difference in the views of GPs and nurses. The factor on which there was closest agreement and which also had the lowest mean score across the two groups (i.e closest to 'strongly agree') was 'poor communication between GPs and nurses will impact on after hours PC service provision'. Another factor on which there was close agreement was 'patients being unwilling to call after hours services when they were available'. These factors have also been identified in earlier studies [20-24].

The three factors on which there was a significant difference of view between the two groups were: nurses feeling unsafe at night, limited access to after hours emergency medication, and limited availability of GPs after hours. In each case the nurses indicated that these issues were more likely to impact on after hours services than did the GPs. There are two likely factors which may have contributed to the difference of views in relation to nurse safety. Nurses are more likely to be focused on that than other work force sectors. Also in this study, as already indicated very few of the GPs personally attended to after hours calls these being addressed by locum services. It is very likely that among the participants of this study nurses were much more likely to be making after hours calls than the GPs. Limited access to emergency medication after hours has also been reported as problematic [22]. There is potential for further investigation of this issue. Are factors such as poor communication, incomplete planning and lack of access to GPs and pharmacies after hours contributing to this problem? As already indicated the use of locum services in the provision of after hours PC, including the issue of cost to patients, needs further investigation. Greater access to GPs after hours was also cited as important by patients in a UK study [25].

### Strategies which may improve after hours PC service provision

A high percentage of both nurses and GPs agreed or strongly agreed that standardised written or individual patient protocols would improve after hours services. It needs to be acknowledged that these are only helpful in relation to after hours care if after hours on call staff, including locums, have access to them.

Of the thirteen suggested strategies for improving after hours PC services there was no significant difference in the views of nurses and GPs in relation to nine of these. Again in each case the nurses agreed more strongly that these strategies would improve after hours services. One of these strategies involved a formal protocol between PC services and aboriginal Australians. Some of the participants (more GPs than nurses) in this study indicated that lack of experience with aboriginal clients prevented them from forming an opinion on this matter.

One important area of significant difference between the two groups was the usefulness of multidisciplinary team meetings, which include the GP, in improving after hours PC services. Possibly GP concerns about the time involved in such meetings and that 49% were unaware that they can use EPC Medicare items to fund this activity, may in part explain this difference. One study has reported that GPs expressed concern about the degree to which they should refer to specialist teams [21]. It is not known if this was a contributing factor to GPs lesser enthusiasm for multidisciplinary team meetings in our study.

Another area of significant difference of view between the two groups related to having more PC trained nurses staffing after hours telephone services. It has been reported [24] that referral and the type of follow-up of triaged after hours calls was related to nurse training and confidence in palliative care. This would support the very strongly held view of nurses that after hours service provision would be improved by increasing the number of PC trained nurses available to these after hours call services.

Support both for carers, either the provision of such opportunities as support groups or respite care provided by PC trained respite carers, or for professionals in the form of great opportunities for debriefing, were other strategies considered. Nurses were significantly more likely to consider that PC trained respite carers would improve after hours service. They were also more likely to consider that service provision would be improved by greater support for staff. This difference may have occurred because participating nurses, who were all members of a PC nurses' special interest group, were much more likely than GPs to be working on a daily basis with people receiving PC and their carers.

The other contentious issue related to changing legislation to allow nurse evaluation of death. Of the thirteen strategies considered this one resulted in the greatest variation in mean scores between the two groups. It is noteworthy that the Victorian Department of Human Services (DHS) has recently (July 2009) issued a Guidance Note [26] relating to the verification of death. This note reinforces that Division 1 and 3 Registered Nurses (as defined by the Health Professionals Registration Act 2005) and Paramedics (as certified by Ambulance Victoria) in an employment context, can verify death, allowing the removal of a body from the site of death. Only registered medical practitioners may certify death by completing the required form.

While all of the issues raised by the participants in this second phase of the project were considered important, it was not possible within the scope of the project to address them all in the final phase, which focused on the production and evaluation of educational tools. For example, some issues such as the shortage of PC trained staff and respite carers cannot be addressed by a project of this nature. However, four main areas, which were considered most feasible in the context of the project, were selected for action. These were: perceived reluctance of patients and carers to utilise available after hours services; poor utilization by GPs of Enhanced Primary Care Medicare Items for palliative care planning and team communication; the importance of advance care planning in relation to appropriate after hours care and communication within multidisciplinary teams which include the GP. A brochure directed to patients and their carers and a DVD, with website version, for health professionals, were produced promoting these main areas. The web address is: .

## Limitations

There are two limitations evident in this study. The very low response rate, particularly from GPs (22%) is clearly an issue in terms of generalisability of the outcomes. Although the response rate of nurses was about twice as high as that of GPs the number of nurse participants was les than half the number of GPs. As already indicated all the nurses had a declared interest in PC where as this was not necessarily an area of interest for the GPs. The decision was made to work with the general GP population as this was thought to be important in getting a clear picture of environment in which community palliative care services are provided. This most likely is a contributing factor to some of the differences of views of the two groups.

It was also evident that there was opportunity for multiple interpretations of some questions. This may well explain the very high level of 'missing' answers to the question relating to the provision of more specific PC beds in local hospitals (see Table 6). It was evident from added comments that some participants considered that the question was not applicable if beds were already provided in their area. The questionnaires will be refined for further use in a larger study.

## Conclusion

This phase of the project identified a number of significant gaps in the provision of after hours care for people receiving palliative care at home. The number of GPs who have completed specialist training in PC remains low with time pressures being a key factor. There is also a notable lack of knowledge on the part of GPs about after hours services available in the community and of the use of EPC items to support planning needs of PC patients. The widespread use of locums in the provision of after hours care also exacerbates provision difficulties and would benefit from further exploration. Communication within multidisciplinary teams, and in particular the inclusion of the GP, remains an inhibitor to the provision of good after hours care. Equitable after hours care in all areas continues to provide a major challenge.

## Competing interests

The authors declare that they have no competing interests.

## Authors' contributions

HMT was involved in data analysis and interpretation, wrote the draft version of this article and has given final approval for the version to be published. MOC was the chief investigator for the project, was involved in the development and design of the project, has revised the article critically for important intellectual content and given final approval for the version to be published. GM was involved in the design and development of the project, has edited and given final approval for the version to be published. BK was involved in the design and development of the project, has edited and given final approval for the version to be published. PS was involved in the design and development of the project, has edited and given final approval for the version to be published.

## Pre-publication history

The pre-publication history for this paper can be accessed here:



## Supplementary Material

Additional File 1 **GP survey questionnaire**. Questionnaire sent to GPs in participating Divisions of General Practice. Click here for file

Additional File 2 **Nurse survey questionnaire**. Questionnaire sent to nurses who are members of Palliative Care Nurses special interest group. Click here for file

## References

[B1] Hudson R, O'Connor M (2007). Palliative Care and Aged Care: A Guide to Practice.

[B2] Allen S, Chapman Y, O'Connor M, Francis K (2008). Discourses associated with nursing aged people who are dying in the Australian context: a review of the literature. Int Nurs Rev.

[B3] Australian Government Department of Health & Ageing (2004). Guidelines for a Palliative Approach in Residential Aged Care: The National Palliative Care Program.

[B4] Kuebler KK, Berry PH, Heidrich DE (2002). End of Life Care: Clinical Practice Guidelines.

[B5] Palmer GR, Short SD (2007). Health Care & Public Policy: An Australian Analysis.

[B6] Boyd CO, Vernon GM (1998). Primary care of the older adult with end-stage Alzheimer's disease. Nurse Practitioner: American Journal of Primary Health Care.

[B7] Walshe C, Caress A, Chew-Graham C, Todd C (2008). Implementation and impact of the Gold Standards Framework in community palliative care: a qualitative study of three primary care trusts. Palliat Med.

[B8] Evans R, Stone D, Elwyn G (2003). Organizing Palliative care for rural populations: a systematic review of the evidence. Fam Pract.

[B9] Farber NJ, Urban SY, Collier VU, Metzger M, Weiner J, Boyer EG (2004). Frequency and perceived competence in providing palliative care to terminally ill patients: a survey of primary care physicians. J Pain Symptom Manage.

[B10] Goldschmidt D, Groenvold M, Johnsen AT, Stromgren AS, Krasnik A, Schmidt L (2005). Cooperating with a palliative home-care team: expectations and evaluations of GPs and district nurses. Palliat Med.

[B11] Jo S, Brazil K, Lohfeld L, Willison (2007). Caregiving at the end of life: Perspectives from spousal caregivers and care recipients. Palliat Support Care.

[B12] Elsey B, McIntyre J (1996). Assessing a supportive and learning network for palliative care workers in a country area of South Australia. Aust J Rural Health.

[B13] Australian Department of Health and Ageing (2000). Enhanced Primary Care Medicare Items: Note A30.

[B14] Zapart S, Kenny P, Servis B, Wiley S (2007). Home-based palliative care in Sydney, Australia: the carer's perspective on the provision of informal care. Health Soc Care Community.

[B15] Philips J, Davidson PM, Newton PJ, DiGiacomo M (2008). Supporting Patients and Their Caregivers After-Hours at the End-of-Life: The Role of Telephone Support. J Pain Symptom Manage.

[B16] Brumley D, Fisher J, Robinson H, Ashby M (2006). Improving access to clinical information in after hours community palliative care. Aust J Adv Nurs.

[B17] Chan SQ, Yong E, Ting A, Kendrick M, DeWitt DE (2007). Rural healthcare provider options about implementation of an after-hours rural telephone triage system. Rural Remote Health.

[B18] Ciechomski L, Tan H, O'Connor M, Miles G, Klein B, Schattner P (2009). After Hours Palliative Care Provision in Rural and urban Victoria, Australia. Asia Pacific Journal of Health Management.

[B19] Rhee J, Zwar N, Vagholkar S, Dennis S, Broadbent A, Mitchell G (2008). Attitude and Barriers to Involvement in Palliative Care by Australian Urban General Practitioners. J Palliat Med.

[B20] Low JA, Liu RK, Strutt R, Chye R (2001). Specialist, community palliative care services - a survey of general practitioners' experiences in Eastern Sydney. Support Care Cancer.

[B21] Mitchell GK (2002). How well do general practitioners deliver palliative care? A systematic review. Palliat Med.

[B22] Worth A, Boyd K, Kendall M, Heaney D, Macleod U, Cormie P, Hockley J, Murray S (2006). Out of hours palliative care: a qualitative study of cancer patients, carers and professionals. Br J Gen Pract.

[B23] Wilkes L, Mohan S, White K, Smith H (2004). Evaluation of an after hours telephone support service for rural palliative care patients and their families: A pilot study. Aust J Rural Health.

[B24] Aranda SK, Hayman-White K, Devilee L, O'Connor M, Bence G (2001). Inpatient hospital triage of 'after-hours' calls to a community palliative care service. Int J Pall Nurs.

[B25] Borgsteed S, Graafland-Riedstra C, Deliens L, Francke A, van Eijk J, Willems D (2006). Good end-of-life care according to patients and their GPs. Br J Gen Pract.

[B26] Victorian Department of Health Services (2009). Verification of Death by Nurses and Paramedics - Guidance Note.

