# Supplementary material for: GP and nurses' perceptions of how after hours care for people receiving palliative care at home could be improved: a mixed methods study
Source: BMC Palliat Care. 2009 Sep 14;8:13. doi: 10.1186/1472-684X-8-13 (PMC2753575; doi:10.1186/1472-684X-8-13)
Supplement: Additional File 1 — GP survey questionnaire. Questionnaire sent to GPs in participating Divisions of General Practice. [file 1472-684X-8-13-S1.doc]

| **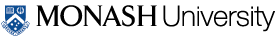** |  | ID Code: P |
| --- | --- | --- |

**What happens after dark? Improving after hours palliative care planning**

**in urban and rural Victoria**

**PART A – YOUR DETAILS**

Please tick the appropriate box

| 1. | Your gender | Female……………………………….. | ❑ | 1 |
| --- | --- | --- | --- | --- |
|  |  | Male…………………………………… | ❑ | 2 |

| 2. | Please write your practice postcode | ❑❑❑❑ |  |
| --- | --- | --- | --- |

| 3. | Number of years in practice | 0 - 5 years | ❑ | 1 |
| --- | --- | --- | --- | --- |
|  |  | 6 – 10 years | ❑ | 2 |
|  |  | 11 – 20 years | ❑ | 3 |
|  |  | > 20 years | ❑ | 4 |

| 4a. | Have you completed postgraduate palliative care training? | Yes……………………………………. | ❑ | 1 |
| --- | --- | --- | --- | --- |
|  |  | No……………………………………... | ❑ | 2 |

| 4b. | If yes, please describe course and year completed | _______________________________________________________________________________________ |
| --- | --- | --- |

| 4c. | If no, select reason why course not undertaken | Lack of available course……………. | ❑ | 1 |
| --- | --- | --- | --- | --- |
|  |  | Lack of time………………………….. | ❑ | 2 |
|  |  | Lack of interest………………………. | ❑ | 3 |
|  |  | Other (specify) _________________ | ❑ | 4 |

**PART B – YOUR PRACTICE**

| 5a. | Do you currently provide after hours palliative care through: | Phone calls to patients | ❑ | 1 |
| --- | --- | --- | --- | --- |
|  |  | Home visits to patients……………… | ❑ | 2 |

| 5b. | If you **do not** **provide after hours palliative care**, please indicate the reason: | Prefer to work during the day………. | ❑ | 1 |
| --- | --- | --- | --- | --- |
|  |  | Family commitments………………… | ❑ | 2 |
|  |  | Lack of financial incentive………….. | ❑ | 3 |
|  |  | Personal safety concerns…………... | ❑ | 4 |
|  |  | Live too far away to provide home visits………………………………….. | ❑ | 5 |
|  |  | Other (specify)_________________ | ❑ | 6 |

| 6. | Do you give your work mobile number to: | Patients receiving palliative care | Yes ❑_1_ | No ❑_2_ |
| --- | --- | --- | --- | --- |
|  |  | GPs providing palliative care | Yes ❑_1_ | No ❑_2_ |
|  |  | Not applicable | Yes ❑_1_ | No ❑_2_ |
| 7. | Do you give your personal mobile number to: | Patients receiving palliative care | Yes ❑_1_ | No ❑_2_ |
|  |  | GPs providing palliative care | Yes ❑_1_ | No ❑_2_ |
|  |  | Not applicable | Yes ❑_1_ | No ❑_2_ |

| 8. | If your service provides after hours palliative care, please indicate the hours and days available: | No of days/week ______ |  |  |
| --- | --- | --- | --- | --- |
|  |  | No of hours/week ______ |  |  |

| 9a. | Do you use the **Enhanced Primary Care** **(EPC)** **Medicare item numbers** (eg. GP care plans) | Yes……………………………………. | ❑ | 1 |
| --- | --- | --- | --- | --- |
|  |  | No……………………………………... | ❑ | 2 |

| 9b. | Are you aware that you can use the **EPCs** for palliative care planning? | Yes……………………………………. | ❑ | 1 |
| --- | --- | --- | --- | --- |
|  |  | No…………………………………….. | ❑ | 2 |

| 9c. | If you do not use the **EPCs,** please indicate the reason: | The remuneration is not very much. | ❑ | 1 |
| --- | --- | --- | --- | --- |
|  |  | Don’t have time to do the paperwork | ❑ | 2 |
|  | | They don’t add anything to patient care………………………… | ❑ | 3 |
|  |  | Other reason (specify)_____________  _____________________________ | ❑ | 4 |

| 10. | When do you usually refer a patient to palliative care services?  ______________________________________________________________________  ______________________________________________________________________ |  |  |
| --- | --- | --- | --- |
|  |  |

**PART C – AFTER HOURS PALLIATIVE CARE IN YOUR REGION**

| 11. | If you **do not provide after hours care**, who provides palliative care after hours services?  (Tick all applicable responses) | My practice partners | ❑ | 1 |
| --- | --- | --- | --- | --- |
|  |  | Doctors from another practice | ❑ | 2 |
|  |  | District nursing | ❑ | 3 |
|  |  | Palliative care service | ❑ | 4 |
|  | | Hospital emergency department | ❑ | 5 |
|  |  | Palliative care inpatient unit………… | ❑ | 6 |
|  |  | Other (specify) _________________ | ❑ | 7 |
|  |  | No one……………………………….. | ❑ | 8 |
|  |  | Don’t know…………………………… | ❑ | 9 |
|  | | Not applicable……………………….. | ❑ | 10 |

| 12. | What type of after hours services are provided? (Tick all applicable responses) | | On-call GP……………………………. | ❑ | 1 |
| --- | --- | --- | --- | --- | --- |
|  |  |  | On-call nurse ………………………... | ❑ | 2 |
|  |  |  | Home visiting by district nurse | ❑ | 3 |
|  |  |  | Home visiting by palliative care service | ❑ | 4 |
|  | | | Other (specify) _________________ | ❑ | 5 |
|  |  |  | No one……………………………….. | ❑ | 6 |
|  |  |  | Don’t know…………………………… | ❑ | 7 |
| 13a. | | Is there a locum service in your region? | Yes……………………………………. | ❑ | 1 |
|  |  |  | No……………………………………... | ❑ | 2 |

| 13b | Does the locum service in your region provide a person that can be contacted for advice after hours? | Yes…………………………………… | ❑ | 1 |
| --- | --- | --- | --- | --- |
|  |  | No…………………………………….. | ❑ | 2 |
|  |  | Don’t know…………………………… | ❑ | 3 |
| 13c | If yes, please describe:________________________________________________________ | | | |
| 14. | Is there a formal **after hours telephone triage protocol** in place in your Region? | Yes | ❑ | 1 |
|  |  | No | ❑ | 2 |
|  |  | Don’t know | ❑ | 3 |
|  |  | N/A | ❑ | 4 |

| 15a. | Is there a particular resource that you find useful in your work with patients who are receiving palliative care? | Yes……………………………………. | ❑ | 1 |
| --- | --- | --- | --- | --- |
|  |  | No……………………………………... | ❑ | 2 |
| 15b. | If yes, please describe:________________________________________________________  __________________________________________________________________________ | | | |
| 15c. | If no, what type of resource would be useful? ______________________________________  __________________________________________________________________________  __________________________________________________________________________ | | | |

16a Please indicate your degree of satisfaction or dissatisfaction with each statement, with regard to **after hours palliative care**, by placing a tick in the appropriate box.

| In my Region: | **Very satisfactory** | **Satisfactory** | **Unsatisfactory** | **Very unsatisfactory** | **Don’t know** |
| --- | --- | --- | --- | --- | --- |
|  | 1 | 2 | 3 | 4 | 5 |
| Hospital discharge planning is: | ❑ | ❑ | ❑ | ❑ | ❑ |
| Home care palliative care planning is: | ❑ | ❑ | ❑ | ❑ | ❑ |
| The current provision of after hours palliative care services is: | ❑ | ❑ | ❑ | ❑ | ❑ |

| 16b. | Please use this space to make any extra comments on after hours palliative care services in your Region: ____________________________________________________________________________________________________________________________________________________________________________________________________________________________________  ____________________________________________________________________________ |
| --- | --- |

**PART D – ABOUT THE AVERAGE PATIENT RECEIVING PALLIATIVE CARE AT HOME**

| 17. | How does the patient manage medications related to palliative care after hours? ____________________________________________________________________________  ____________________________________________________________________________  ____________________________________________________________________________  ____________________________________________________________________________ |
| --- | --- |

| 18a. | If one of your patients requested an unplanned after hours visit from you, are you able to attend? | Always | ❑ | 1 |
| --- | --- | --- | --- | --- |
|  |  | In most cases | ❑ | 2 |
|  |  | Sometimes | ❑ | 3 |
|  |  | Never | ❑ | 4 |
|  | | Not applicable………………………. | ❑ | 5 |

| 18b. | If you or someone from your practice cannot attend after hours home visits, how is this managed? ____________________________________________________________________________  ____________________________________________________________________________  ____________________________________________________________________________  ____________________________________________________________________________ |
| --- | --- |

| 19. | If any of your patients receiving palliative care at home dies, are you notified? | Always | ❑ | 1 |
| --- | --- | --- | --- | --- |
|  |  | In most cases | ❑ | 2 |
|  |  | Sometimes | ❑ | 3 |
|  |  | Never | ❑ | 4 |
|  | | Not applicable………………………. | ❑ | 5 |

**PART E – FACTORS WHICH MAY AFFECT AFTER HOURS PALLIATIVE CARE**

20a . We would like to know your views on factors which may affect after hours palliative care. Please **rate your level of agreement or disagreement** with each of the following statements.

| After hours palliative care service delivery may be less than optimal if: | **Strongly Agree** | **Agree** | **Disagree** | **Strongly Disagree** | **Don’t know** |
| --- | --- | --- | --- | --- | --- |
|  | 1 | 2 | 3 | 4 | 5 |
| Communication between nurses and GPs is not regular. | ❑ | ❑ | ❑ | ❑ | ❑ |
| The cost of locum services for patients is too high. | ❑ | ❑ | ❑ | ❑ | ❑ |
| Patients are not willing to call after hours services, e.g., because they do not wish to wake or disturb the nurse or GP on call. | ❑ | ❑ | ❑ | ❑ | ❑ |
| Nurses are unsafe at night. | ❑ | ❑ | ❑ | ❑ | ❑ |
| There is limited mobile phone coverage. | ❑ | ❑ | ❑ | ❑ | ❑ |
| Access to emergency medication after hours is restricted. | ❑ | ❑ | ❑ | ❑ | ❑ |
| There is a limited number of GPs doing after hours palliative care home visits. | ❑ | ❑ | ❑ | ❑ | ❑ |
| There is a limited bank of nursing staff for after hours palliative care. | ❑ | ❑ | ❑ | ❑ | ❑ |
| After hours staff cannot access an interpreter after hours. | ❑ | ❑ | ❑ | ❑ | ❑ |

| 20b | Please elaborate on any of the above factors which may affect after hours palliative care or describe any other factors which may affect after hours palliative care in your Region:  _____________________________________________________________________________  __________________________________________________________________________________________________________________________________________________________  _____________________________________________________________________________  _____________________________________________________________________________ |
| --- | --- |

**PART F – STRATEGIES WHICH MAY IMPROVE AFTER HOURS PALLIATIVE CARE**

21a. We would like to know your views on strategies which may improve after hours palliative care. Please indicate how much you **agree or disagree** with each of the following statements by placing a tick in the appropriate box.

| After hours palliative care service delivery might be improved with: | **Strongly Agree** | **Agree** | **Disagree** | **Strongly Disagree** | **Don’t know** | **Not applicable** |
| --- | --- | --- | --- | --- | --- | --- |
|  | 1 | 2 | 3 | 4 | 5 | 6 |
| A standardised written referral protocol explaining  - after hours management,  - contact details of service providers, and  - medication regime and supply. | ❑ | ❑ | ❑ | ❑ | ❑ |  |
| An individualised patient protocol covering instructions for resuscitation, access to medications and emergency afters care plans. | ❑ | ❑ | ❑ | ❑ | ❑ |  |
| A formal protocol between palliative care service and indigenous Australian communities, to ensure cultural sensitivity in palliative care. | ❑ | ❑ | ❑ | ❑ | ❑ |  |
| Regular meetings between nurses /case managers /agencies and GPs to discuss specific palliative care patients and issues. | ❑ | ❑ | ❑ | ❑ | ❑ |  |
| Referral to palliative care services at the time of diagnosis of a life limiting illness. | ❑ | ❑ | ❑ | ❑ | ❑ |  |
| More nurses for after hours home visits. | ❑ | ❑ | ❑ | ❑ | ❑ |  |
| Palliative care trained nurse on-call for telephone advice. | ❑ | ❑ | ❑ | ❑ | ❑ |  |
| Palliative care trained respite carers. | ❑ | ❑ | ❑ | ❑ | ❑ |  |
| More support and debriefing for GPs. | ❑ | ❑ | ❑ | ❑ | ❑ |  |
| More support for carers e.g., support groups. | ❑ | ❑ | ❑ | ❑ | ❑ |  |
| Specific palliative care beds in local hospitals (please select not applicable if there already beds in your area). | ❑ | ❑ | ❑ | ❑ | ❑ | ❑ |
| Greater access to equipment for the home e.g., hospital beds. | ❑ | ❑ | ❑ | ❑ | ❑ |  |
| Legislative change so that nurses can evaluate extinction of life. | ❑ | ❑ | ❑ | ❑ | ❑ |  |

| 21b | Please elaborate on any of the above strategies which may improve after hours palliative care, or list any other strategies that you believe would improve after hours care in your region:  _____________________________________________________________________________  __________________________________________________________________________________________________________________________________________________________  _____________________________________________________________________________  _____________________________________________________________________________ |
| --- | --- |

Please feel free to use the space below to add any additional comments you wish to make about after hours palliative care in your region.

.

# Code P

| **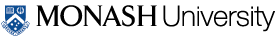** |  |  |
| --- | --- | --- |

From the Palliative Care Research Team at Monash University, thank you very much for completing this survey. Please post it back in the addressed envelope supplied by **Friday 16^th^ November 2007**. Once we receive your questionnaire, you will go into the draw to win a double movie pass and one night’s accommodation in Melbourne.

Please supply your contact details below and return this page with your questionnaire. ☺

Your Name: …………………………………………………………………..

Your postal address: …………………………………………………………………..

…………………………………………………………………..

…………………………………………………………………..

Your email address: …………………………………………………………………..

A contact number: …………………………………………………………………..
