# Supplementary material for: GP and nurses' perceptions of how after hours care for people receiving palliative care at home could be improved: a mixed methods study
Source: BMC Palliat Care. 2009 Sep 14;8:13. doi: 10.1186/1472-684X-8-13 (PMC2753575; doi:10.1186/1472-684X-8-13)
Supplement: Additional File 2 — Nurse survey questionnaire. Questionnaire sent to nurses who are members of Palliative Care Nurses special interest group. [file 1472-684X-8-13-S2.doc]

| **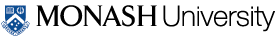** |  | ID Code: RDNSR |
| --- | --- | --- |

**What happens after dark? Improving after hours palliative care planning in urban and rural Victoria**

**PART A – YOUR DETAILS**

Please tick the appropriate box

| 1. | Gender | Female | ❑ | 1 |
| --- | --- | --- | --- | --- |
|  |  | Male | ❑ | 2 |

| 2. | Please write your work location postcode | ❑❑❑❑ |  |
| --- | --- | --- | --- |

| 3. | Number of years in nursing | 0 - 5 years | ❑ | 1 |
| --- | --- | --- | --- | --- |
|  |  | 6 – 10 years | ❑ | 2 |
|  |  | 11 – 20 years | ❑ | 3 |
|  |  | > 20 years | ❑ | 4 |

| 4a. | Have you completed a formal palliative care course? | Yes……………………………………. | ❑ | 1 |
| --- | --- | --- | --- | --- |
|  |  | No……………………………………... | ❑ | 2 |

| 4b. | If yes, please describe course and year completed | _______________________________________________________________________________________ |
| --- | --- | --- |

| 4c. | If no, select reason why course not undertaken | Lack of available course | ❑ | 1 |
| --- | --- | --- | --- | --- |
|  |  | Lack of time | ❑ | 2 |
|  |  | Lack of interest | ❑ | 3 |
|  |  | Other (specify) ________________ | ❑ | 4 |

**PART** B – YOUR SERVICE

| 5a. | Does your service currently provide after hours palliative care through: | Phone calls to patients | ❑ | 1 |
| --- | --- | --- | --- | --- |
|  |  | Home visits to patients……………… | ❑ | 2 |

| 5b. | If you **do not** **provide after hours palliative care**, please indicate the reason: | Prefer to work during the day………. | ❑ | 1 |
| --- | --- | --- | --- | --- |
|  |  | Family commitments………………… | ❑ | 2 |
|  |  | Lack of financial incentive………….. | ❑ | 3 |
|  |  | Personal safety concerns…………... | ❑ | 4 |
|  |  | Other (specify)__________________  _____________________________  _____________________________  _____________________________ | ❑ | 5 |

| 6. | Do you give your work mobile number to: | Patients receiving palliative care | Yes ❑_1_ | No ❑_2_ |
| --- | --- | --- | --- | --- |
|  |  | GPs providing palliative care | Yes ❑_1_ | No ❑_2_ |
|  |  | Not applicable | Yes ❑_1_ | No ❑_2_ |

| 7. | Do you give your personal mobile number to: | Patients receiving palliative care | Yes ❑_1_ | No ❑_2_ |
| --- | --- | --- | --- | --- |
|  |  | GPs providing palliative care | Yes ❑_1_ | No ❑_2_ |
|  |  | Not applicable | Yes ❑_1_ | No ❑_2_ |

| 8. | If your service provides after hours palliative care, please indicate the hours and days available: | No of days/week ______ |  |  |
| --- | --- | --- | --- | --- |
|  |  | No of hours/week ______ |  |  |

**PART C – AFTER HOURS PALLIATIVE CARE IN YOUR REGION**

| 9. | Who else provides after hours services in your region? Circle all applicable responses | District nursing | ❑ | 1 |
| --- | --- | --- | --- | --- |
|  |  | General practitioner | ❑ | 2 |
|  |  | Palliative care service | ❑ | 3 |
|  |  | Hospital emergency department | ❑ | 4 |
|  | | Palliative care inpatient unit………… | ❑ | 5 |
|  |  | Other (specify) _________________ | ❑ | 6 |
|  |  | No one………………………………... | ❑ | 7 |
|  |  | Don’t know…………………………… | ❑ | 8 |

| 10. | What type of after hours services are provided? Circle all applicable responses | Home visiting by palliative care program | ❑ | 1 |
| --- | --- | --- | --- | --- |
|  |  | Home visiting by district nurse | ❑ | 2 |
|  |  | On-call GP with telephone | ❑ | 3 |
|  |  | On-call nurse with telephone | ❑ | 4 |
|  | | Other (specify) _________________ | ❑ | 5 |
|  |  | None………………………………... | ❑ | 6 |
|  |  | Don’t know…………………………… | ❑ | 7 |

| 11. | Is there a locum service in your region? | Yes……………………………………. | ❑ | 1 |
| --- | --- | --- | --- | --- |
|  |  | No……………………………………... | ❑ | 2 |

| 12a. | Does the locum service in your region provide a person that can be contacted for advice after hours? | Yes……………………………………. | ❑ | 1 |
| --- | --- | --- | --- | --- |
|  |  | No……………………………………... | ❑ | 2 |

| 12b. | If yes, please describe: _____________________________________________________________________ |
| --- | --- |

| 13a. | Is there a formal **after hours telephone triage protocol** in place in your region? | Yes……………………………………. | ❑ | 1 |
| --- | --- | --- | --- | --- |
|  |  | No……………………………………... | ❑ | 2 |
|  |  | Don’t know…………………………… | ❑ | 3 |
|  |  | N/A……………………………………. | ❑ | 4 |

| 13b. | If yes, please describe the after hours telephone triage protocol: ____________________________________________________________________________  ____________________________________________________________________________  ____________________________________________________________________________  ____________________________________________________________________________ |
| --- | --- |

| 14a. | Is there a particular resource that you find useful in your work with patients who are receiving palliative care? | Yes……………………………………. | ❑ | 1 |
| --- | --- | --- | --- | --- |
|  |  | No……………………………………... | ❑ | 2 |
| 14b. | If yes, please describe:________________________________________________________  ___________________________________________________________________________  ___________________________________________________________________________ | | | |
| 14c. | If no, what type of resource would be useful? ___________________________________________________________________________­­­­­­­­­___________________________________________________________________________ | | | |

15a. Please indicate your degree of satisfaction or dissatisfaction with each statement, with regard to **after hours palliative care**, by placing a tick in the appropriate box.

| In my Region: | **Very satisfactory** | **Satisfactory** | **Unsatisfactory** | **Very unsatisfactory** | **Don’t know** |
| --- | --- | --- | --- | --- | --- |
|  | 1 | 2 | 3 | 4 | 5 |
| Hospital discharge planning is: | ❑ | ❑ | ❑ | ❑ | ❑ |
| Home care palliative care planning is: | ❑ | ❑ | ❑ | ❑ | ❑ |
| The current provision of after hours palliative care services is: | ❑ | ❑ | ❑ | ❑ | ❑ |

| 15b. | Please comment on after hours care in your region: ____________________________________________________________________________________________________________________________________________________________________________________________________________________________________  ____________________________________________________________________________  ____________________________________________________________________________ |
| --- | --- |

**PART D –ABOUT THE PATIENT/FAMILY RECEIVING PALLIATIVE CARE AT HOME**

| 16. | How does the patient/family manage medication after hours? ____________________________________________________________________________  ____________________________________________________________________________  ____________________________________________________________________________  ____________________________________________________________________________ |
| --- | --- |

| 17a. | If one of your patients/family requested an unplanned visit from your service, is the service able to attend? | Always | ❑ | 1 |
| --- | --- | --- | --- | --- |
|  |  | In most cases | ❑ | 2 |
|  |  | Sometimes | ❑ | 3 |
|  |  | Never | ❑ | 4 |
|  | | Not applicable………………………. | ❑ | 5 |

| 17b. | If your service is unable to attend after hours, how is this managed? ____________________________________________________________________________  ____________________________________________________________________________  ________________________________________________________________________________________________________________________________________________________  ____________________________________________________________________________ |
| --- | --- |

| 18. | If one of your patients who is receiving palliative care at home dies, and you are not present at the time of death, are you notified? | Always | ❑ | 1 |
| --- | --- | --- | --- | --- |
|  |  | In most cases | ❑ | 2 |
|  |  | Sometimes | ❑ | 3 |
|  |  | Never | ❑ | 4 |
|  | | Not applicable………………………. | ❑ | 5 |

**PART E – FACTORS WHICH MAY AFFECT AFTER HOURS PALLIATIVE CARE**

19a. We would like to know your views on factors which may affect after hours palliative care. Please indicate how much you **agree or disagree** with each of the following statements by placing a tick in the appropriate box.

| After hours palliative care service delivery may be less than optimal if: | **Strongly Agree** | **Agree** | **Disagree** | **Strongly Disagree** | **Don’t know** |
| --- | --- | --- | --- | --- | --- |
|  | 1 | 2 | 3 | 4 | 5 |
| Communication between nurses and GPs is not regular. | ❑ | ❑ | ❑ | ❑ | ❑ |
| The cost of locum services for patients is too high. | ❑ | ❑ | ❑ | ❑ | ❑ |
| Patients are not willing to call after hours services e.g., because they do not wish to wake or disturb the nurse on call or GP. | ❑ | ❑ | ❑ | ❑ | ❑ |
| Nurses are unsafe at night. | ❑ | ❑ | ❑ | ❑ | ❑ |
| There is limited mobile phone coverage. | ❑ | ❑ | ❑ | ❑ | ❑ |
| Access to emergency medication after hours is restricted. | ❑ | ❑ | ❑ | ❑ | ❑ |
| There is a limited number of GPs doing after hours palliative care home visits. | ❑ | ❑ | ❑ | ❑ | ❑ |
| There is a limited bank of nursing staff to provide after hours palliative care. | ❑ | ❑ | ❑ | ❑ | ❑ |
| After hours staff cannot access to an interpreter. | ❑ | ❑ | ❑ | ❑ | ❑ |

| 19b | Please elaborate on the above factors which may affect after hours palliative care, or describe other factors which may affect after hours palliative care in your region:  _____________________________________________________________________________  __________________________________________________________________________________________________________________________________________________________  __________________________________________________________________________________________________________________________________________________________  _____________________________________________________________________________  _____________________________________________________________________________ |
| --- | --- |

**PART F – STRATEGIES TO IMPROVE AFTER HOURS PALLIATIVE CARE**

20a. We would like to know your views on strategies which may improve after hours palliative care. Please indicate how much you **agree or disagree** with each of the following statements by placing a tick in the appropriate box.

| After hours palliative care service delivery might be improved with: | **Strongly Agree** | **Agree** | **Disagree** | **Strongly Disagree** | **Don’t know** | **Not applicable** |
| --- | --- | --- | --- | --- | --- | --- |
|  | 1 | 2 | 3 | 4 | 5 | 6 |
| A standardised written referral protocol explaining  - after hours management,  - contact details of service providers, and  - medication regime and supply. | ❑ | ❑ | ❑ | ❑ | ❑ |  |
| An individualised patient protocol covering instructions for resuscitation, access to medications and emergency after hours care plan. | ❑ | ❑ | ❑ | ❑ | ❑ |  |
| A formal protocol between palliative care service and indigenous Australian communities, to ensure cultural sensitivity in palliative care. | ❑ | ❑ | ❑ | ❑ | ❑ |  |
| Regular meetings between nurses /case managers /agencies and GPs to discuss specific palliative care patients and issues. | ❑ | ❑ | ❑ | ❑ | ❑ |  |
| Referral to palliative care services at the time of diagnosis of a life limiting illness. | ❑ | ❑ | ❑ | ❑ | ❑ |  |
| More nurses for after hours home visits. | ❑ | ❑ | ❑ | ❑ | ❑ |  |
| Palliative care trained nurse on-call for telephone advice. | ❑ | ❑ | ❑ | ❑ | ❑ |  |
| Palliative care trained respite carers. | ❑ | ❑ | ❑ | ❑ | ❑ |  |
| More support and debriefing for nurses. | ❑ | ❑ | ❑ | ❑ | ❑ |  |
| More support for carers eg. support groups | ❑ | ❑ | ❑ | ❑ | ❑ |  |
| Specific palliative care beds in local hospitals (please select not applicable if there already beds in your area). | ❑ | ❑ | ❑ | ❑ | ❑ | ❑ |
| A hospice in your local area (please select not applicable if there is a hospice in your area). | ❑ | ❑ | ❑ | ❑ | ❑ | ❑ |
| Greater access to equipment for the home eg. hospital beds. | ❑ | ❑ | ❑ | ❑ | ❑ |  |
| Greater use of the unassigned bed fund to provide equipment in the home. | ❑ | ❑ | ❑ | ❑ | ❑ |  |
| Legislative change so that nurses can evaluate extinction of life. | ❑ | ❑ | ❑ | ❑ | ❑ |  |
| Multidisciplinary management team. | ❑ | ❑ | ❑ | ❑ | ❑ |  |

| 20b | Please elaborate on the above strategies which may improve after hours palliative care, or list other strategies that you believe would improve after hours care in your region:  _____________________________________________________________________________  __________________________________________________________________________________________________________________________________________________________  _____________________________________________________________________________  __________________________________________________________________________________________________________________________________________________________ |
| --- | --- |

21. Please indicate which of the following issues could be managed after hours through a phone consultation, and/or a home visit. Please tick all boxes that apply.

|  | Phone call | Home visit |
| --- | --- | --- |
| Patient and or carer anxiety | ❑ | ❑ |
| Advice regarding pain | ❑ | ❑ |
| Advice regarding symptom management | ❑ | ❑ |
| Problems with equipment | ❑ | ❑ |
| Query regarding imminent death | ❑ | ❑ |
| Patient has died | ❑ | ❑ |
| Emergency eg. sudden deterioration in health | ❑ | ❑ |
| Other (please specify) _______________________________________  _____________________________________________________________________________________________________________________ | ❑ | ❑ |

Please feel free to use the space below to elaborate on after hours palliative care in your region.
